# Health and Environment Information Systems for Exposure and Disease Mapping, and Risk Assessment

**DOI:** 10.1289/ehp.6736

**Published:** 2004-04-15

**Authors:** Lars Jarup

**Affiliations:** Small Area Health Statistics Unit, Department of Epidemiology and Public Health, Imperial College London, London, United Kingdom

**Keywords:** disease mapping, exposure assessment, GIS, health and environment information systems, risk assessment, spatial epidemiology

## Abstract

A large number of chemicals are used on a regular basis in modern society. Thousands of new chemicals are added each year, many of which may have toxic properties constituting potential health hazards. Rapid assessment of the risk associated with the use of these chemicals is therefore essential to protect people from exposure to potentially harmful substances. Exposures to chemicals (and physical agents) are typically unevenly distributed geographically as well as temporally. Disease occurrence also shows geographically varying patterns. Geographic information systems (GIS) may be used to produce maps of exposure and/or disease to reveal spatial patterns. Exposure mapping using advanced GIS modeling may enhance exposure assessment in environmental epidemiology studies. Disease maps can be valuable tools in risk assessment to explore changes in disease patterns potentially associated with changes in environmental exposures. Spatial variations in risk and trends related to distance from pollution sources may be studied using software tools such as the Rapid Inquiry Facility, developed by the U.K. Small Area Health Statistics Unit and enhanced in the European Health and Environment Information System project, for an initial quick evaluation of any potential health hazards associated with an environmental pollutant.

Geographic information systems (GIS) are being used increasingly in environmental epidemiology, adding a further dimension to risk assessment (Vine et al. 1997).

A thorough exposure assessment is vitally important to risk assessment. Because measurements of environmental contaminants are expensive, few geographic monitoring locations are normally used. For example, air pollutants are usually measured in one or a few places in a city or a region, even though air pollution is not evenly distributed. Consequently, modeled pollution data are commonly used to assess exposure.

Accurate and detailed health data are equally important for risk assessment. Mortality and cancer incidence are often well reported and of good quality, whereas other health data (such as congenital anomalies and hospital admissions) may be underascertained and of uneven quality. The reporting of a health outcome may vary between geographic regions and over time. Thus, although geographic patterns of disease may be used to infer possible associations between environmental pollutants and health effects, such patterns may also reflect differences in health data recording.

Overlaying maps of exposure and populations may define populations at risk. However, linking of exposure and disease is highly dependent on the accuracy of exposure assessment as well as the time elapsed between initial exposure and disease (the latency time). The longer the latency time, the more difficult it will be to associate exposure with disease because of changes in exposure over time and/or population changes due to migration.

In spite of the difficulties of dealing with spatial data, risk assessment may benefit greatly from a spatial approach, as demonstrated by the articles in this mini-monograph. Indeed, it has been argued that “risk professionals will not mislead by presenting maps—they mislead by not presenting maps” (Hargrove et al. 1996).

## Exposure Mapping

The role of GIS in exposure assessment is discussed in detail by Nuckols et al. (2004). As pointed out by the authors, it is important to keep in mind the definition of exposure. A person is considered exposed to an environmental agent if the agent in question has been in contact with a body surface. Nevertheless, sometimes exposure has occurred merely because the supposedly exposed population is living or has lived in a contaminated environment, without demonstrating that contact between the pollutants and the population has occurred. Such careless use of the term “exposure” should be avoided.

For example, soil may be contaminated with chemical waste such as heavy metals and other persistent substances, and exposure may indeed occur if the soil is used for growing vegetables (Staessen et al. 1994) or if there is a leakage of chemicals to groundwater, which may pollute drinking water wells. GIS have been used to map soil contamination, particularly, heavy metals. However, most residents in the contaminated area will not be exposed, and thus such GIS-derived data should not be used indiscriminately to assess exposure.

GIS are commonly used for assessing exposure to air pollution. There is almost always contact between the air pollutant and the human respiratory system, and thus exposure will indeed take place. However, individual exposure may vary greatly depending on many different circumstances such as the proportion of time spent indoors and outdoors, respectively.

Several studies have been performed around point sources of pollution, such as industrial plants, using circular areas at different distances from the source to define exposure zones (e.g., Aylin et al. 2001; Wilkinson et al. 1997). This approach has proven useful although it provides a rather crude estimate of exposure that does not consider, for example, meteorologic conditions or topography

Most studies of the relationship between traffic-related pollution and respiratory disease have used distance from roads as the exposure indicator (e.g., Hoek et al. 2002; Wilkinson et al. 1999). More recently, regression or dispersion modeling using GIS has been used to assess air pollution exposure (e.g., Bellander et al. 2001; Brauer et al. 2003; Briggs et al. 2000).

Similarly, Poulstrup and Larsen (2004) used dispersion modeling to define populations exposed to airborne dioxin around industrial plants in Denmark.

Verkasalo et al. (2004) used distance from a contaminated river as a proxy for exposure to dioxin. The authors note that the use of a nonspecific surrogate measure for exposure may have introduced considerable measurement error or confounding by correlated exposures. Nevertheless, they found the approach useful for an initial assessment of a potential environmental health hazard.

## Disease Mapping

Disease mapping may be used to identify possible disease clusters, to define and monitor epidemics, to provide baseline data on health patterns, and to show changes in disease patterns over time. Disease mapping may also be useful for initial exploration of relationships between exposure and disease, particularly, acute health effects.

Maps of cancer incidence and mortality, computed on a global scale by the International Agency for Research of Cancer, are readily interpretable (Ferlay et al. 2001). Other large-scale maps such as the atlases of cancer mortality produced by the U.S. National Cancer Institute are also relatively easy to interpret (Cancer Mortality Maps & Graphs 1999).

Small-area maps of disease are much more difficult to produce and interpret in a meaningful way. A recent study of prostate cancer did not show any marked geographic variability in incidence at a small-area scale, arguing against a geographically varying, etiologically strong environmental risk factor (Jarup et al. 2002a). However, caution needs to be exercised in the interpretation because of factors such as latency time and migration.

## Risk Assessment

Health and environment information systems based on GIS may be useful in the risk assessment process (for exposure assessment, for disease mapping, for assessing health risks associated with point sources of pollution, and for estimating the numbers of people at risk). The user should be aware of both the strengths and weaknesses associated with this approach.

Studies of variations in risk with distance from pollution sources such as industrial plants (Aylin et al. 2001) or landfill sites (Elliott et al. 2001; Jarup et al. 2002b) have been relatively common in environmental epidemiology.

Attempts to assess risk by overlaying maps of exposure and disease, given the (in)accuracy of the exposure estimates, latency periods, and migration problems, are likely to be misleading and should be avoided.

A main advantage in using GIS for exposure assessment is the possibility of modeling exposure geographically so that individual exposure may be estimated without the need for time-consuming and expensive measurements. Modeling uncertainties must be considered, especially when applied to large areas in particular, as the spatial resolution and coverage of environmental data are often poor.

GIS techniques can be used to estimate number of expected cases in a population potentially exposed to air pollution, by combining dispersion models with demographic data to produce estimates of the number of people exposed to certain levels of air pollution. Existing data on exposure–response relationships can then be used to compute the number of expected cases at each exposure level. Exposure–response relationships are currently available for only a few pollutants (e.g., PM_10_) for which good exposure models are not available, whereas data on exposure–response relationships are sparse for air pollutants, which can be more readily modeled (e.g., nitrogen dioxide)

Disease maps can be useful in risk assessment by defining a baseline pattern of disease that could be followed up by continued mapping of disease occurrence over time to explore potential changes in disease patterns that may be associated with changes in environmental exposures.

Disease mapping is used increasingly to describe variations of disease (most commonly cancer) between regions (Buntinx et al. 2003; Jarup et al. 2002a). Disease mapping may be very valuable as a means of assessing geographic differences in health, but several pitfalls need to be considered.

Arbitrary boundaries (usually administrative areas) are often used to map diseases. If the results are sensitive to change in boundaries, it is obvious that caution should be exercised when interpreting apparent associations between environmental exposures and health effects. This problem has been termed the modifiable area unit problem (Openshaw 1984) and is fundamental in all attempts to map disease using aggregated statistics.

It is clear that administrative boundaries may not be ideal for mapping health outcomes and that choice of boundaries may have a major influence on the results. The use of arbitrary but uniform boundaries such as grid squares may reduce these problems to some extent.

Standardized mortality (morbidity) ratios (SMRs) are commonly used as estimates of the relative risk. A criticism sometimes raised against mapping SMRs is that SMRs are not directly comparable, as they are not based on the same standard population (Julious et al. 2001). This is theoretically correct, but in practice, comparisons of SMRs between geographical areas will be misleading only if the age and sex structure of the populations are extremely disparate (Goldman and Brender 2000), which very rarely occurs in practice. The imprecision of alternative statistical estimates such as directly standardized rate ratios, when calculated on small area scale, is a far more serious problem (Jarup and Best 2003).

## Small Area Health Statistics Unit

The U.K. Small Area Health Statistics Unit (SAHSU) was established in 1987 after a recommendation of an inquiry into the incidence of leukemia in children and young adults near the Sellafield nuclear plant.

The primary purpose of SAHSU is to assess environmental health risks using routinely collected health statistics data. SAHSU has developed a tool, the Rapid Inquiry Facility (RIF), for rapid assessment of environmental health hazards. The RIF produces estimated relative risks for any given condition for the population within defined areas around a point source, relative to the population in a local reference region (Aylin et al. 1999). The system creates maps to illustrate disease variation across small areas, as well as smoothed small-area maps that account for sampling variability in the observed data, to aid interpretation of the results.

Performing substantial research studies is an integral part of SAHSU work, making efficient use of routinely collected data and enhancing these data sets in the process. In recent years special attention has been given to exposure assessment, using advanced GIS techniques to define exposed populations (e.g., Elliott et al. 2001). State-of-the-art statistical methods are of crucial importance for studies of small-area variations in health, and thus there is an ongoing development of such methods within SAHSU (Richardson et al. 2004). Several aspects of spatial epidemiology have recently been reported by SAHSU in a comprehensive book (Elliott et al. 2000).

## European Health and Environment Information System

The European Health and Environment Information System (EUROHEIS ) project was launched in 1999 to improve the understanding of the links between environmental exposures, health outcome, and risk through development of integrated information systems for rapid assessment of relationships between the environment and health at a geographic level.

The European Commission funded EUROHEIS for 3 years (2000–2003). During the first year of the project, the feasibility of implementing an analysis tool in different European countries, based on the SAHSU RIF, was examined. In the Nordic countries (Denmark, Finland, Sweden), health and population data are available at an individual level. In the United Kingdom and the Netherlands, data are available at postcode level, each post-code comprising approximately 15 households in the United Kingdom, and an average of 17 addresses in the Netherlands. The data resolution in Italy and Spain is much lower (municipality level, varying from a few hundred to several thousand people or more). In spite of the differences in data resolution between countries, the feasibility study showed that RIF implementation should be possible in all partner countries, and further development of the RIF was carried out to facilitate the transfer.

During the second year of the project, the RIF was installed in Spain and Sweden, confirming that it was feasible to run the system in countries with widely different data resolution. A similar system was already in place in Finland, whereas a modified version was used in Denmark, taking full advantage of the Danish personal identification system to follow up populations exposed to environmental agents.

In the third year of the project, the usefulness of the system in answering questions concerning environmental health risks was demonstrated through a series of case studies carried out within partner countries. In Denmark, cancer occurrence in a population exposed to airborne dioxins was studied using modeled dioxin emissions, which defined exposure at various distances around the plant (Poulstrup and Larsen 2004). The Finnish partner investigated cancer risk possibly associated with exposure to chlorophenols and polychlorinated dibenzo-*p*-dioxins and poly-chlorinated dibenzofurans emanating from a heavily contaminated river (Verksalo et al. 2004), and in Spain, the RIF was used to study relationships between drinking water hardness and cardiovascular and cerebrovascular mortality, using health and water quality data at municipality level (Ferrandiz et al. 2004).

To disseminate the EUROHEIS project results and to stimulate interest for spatial epidemiology, an international conference was held in Sweden in March 2003, attracting approximately 100 delegates, mainly from Europe but also from Peru, India, Israel, Canada, and the United States. Although the conference focused on the role of EUROHEIS in environmental health, it also comprised a wide range of presentations on exposure assessment, statistical methods, and other aspects of spatial epidemiology. This mini-monograph contains a selection of papers based on presentations at the conference.

## Future Developments

Using concentric circles to identify exposed populations usually leads to a bias of the relative risk toward the null. A better approach would be to use data on wind direction and speed, and temperature as well as local topography to model the pollutant dispersion (Williams and Ogston 2002). The modeled data preferably should be validated by monitoring environmental media (air, water, soil). The potential to incorporate such model output (e.g., from the Atmospheric Dispersion Modeling System) in the RIF would greatly enhance the software.

Maps of relative risks show point estimates almost exclusively and do not consider the uncertainty in the relative risk estimates. Even if confidence intervals for the relative risk estimates are given in accompanying tables, it should be recognized that maps are far more powerful than tables for conveying information about geographic variations in risk. Therefore, techniques need to be developed to also map information about uncertainty in risk estimates in a way that is easy to interpret. A possible solution when using Bayesian smoothing methods is to map the posterior probability of the relative risk of any area exceeding a prespecified threshold (Jarup et al. 2002a; Jarup and Best 2003).

Exposure is commonly based on current environmental data, whereas information on historical exposure tends to be sparse. Many study areas are also prone to extensive migration, and most chronic diseases have long latency times. Therefore, methods should be developed to explore the effects on risk due to migration, as suggested in the article by Elliott and Wartenberg (2004). Some countries such as Denmark may use their unique personal identification system to follow up exposed populations to get more accurate estimates of the relative risk.

Future developments also include translation of the RIF software into a more user-friendly system, making it easily available to additional countries, and facilitation of dissemination via the Internet. Further methodologic progress will make it possible to input data from dispersion (and similar) models to enhance exposure assessment and to develop data export facilities to make further, more advanced statistical analysis possible when needed.
